# Efficacy of bevacizumab and chemotherapy in the first-line treatment of metastatic colorectal cancer: broadening KRAS-focused clinical view

**DOI:** 10.1186/s12876-015-0266-6

**Published:** 2015-03-24

**Authors:** Beatrix Bencsikova, Zbynek Bortlicek, Jana Halamkova, Lenka Ostrizkova, Igor Kiss, Bohuslav Melichar, Tomas Pavlik, Ladislav Dusek, Dalibor Valik, Rostislav Vyzula, Lenka Zdrazilova-Dubska

**Affiliations:** 1 Department of Complex Oncology Care, Masaryk Memorial Cancer Institute, Brno, Czech Republic; 2 Regional Centre for Applied Molecular Oncology, Masaryk Memorial Cancer Institute, Brno, Czech Republic; 3 Institute of Biostatistics and Analyses, Faculty of Medicine, Masaryk University, Brno, Czech Republic; 4 Department of Internal Medicine and Hematooncology, University Hospital Brno, Brno, Czech Republic; 5 Department of Oncology, Palacky University Medical School and Teaching Hospital, Olomouc, Czech Republic; 6 Department of Laboratory Medicine, Masaryk Memorial Cancer Institute, Zluty kopec 7, Brno, 656 53 Czech Republic; 7 Department of Pharmacology, Faculty of Medicine, Masaryk University, Brno, Czech Republic

**Keywords:** Colorectal cancer, Angiogenesis inhibitors, Bevacizumab, KRAS, Biomarkers, Antineoplastic agents, Clinical practice

## Abstract

**Background:**

The aim of the present retrospective study was to analyze clinical outcome and risk factors associated with treatment outcomes according to KRAS status in patient with metastatic colorectal cancer (mCRC) treated with bevacizumab (bev) plus chemotherapy in the first-line setting.

**Methods:**

We performed observational study on 1622 patients with mCRC treated with bev plus oxaliplatin- or irinotecan-based chemotherapy, and correlated treatment outcomes with KRAS mutation status. The primary endpoint was progression-free survival (PFS) and additionally overall survival (OS). Adverse events of bevacizumab and risk factors including location of metastases were evaluated.

**Results:**

Mutation in KRAS was present in 40.6% of mCRC cases. The median PFS in patients with wild-type KRAS (wtKRAS) vs mutant KRAS was 11.5 vs 11.4 months, respectively. The median OS was 30.7 vs 28.4 months (p = 0.312). Patients with KRAS mutation had lung metastases more frequently than wtKRAS individuals (32.0% vs 23.8%; p = 0.001). We observed no difference in clinical outcome between hepatic and extrahepatic metastatic disease.

**Conclusion:**

KRAS mutation does not interfere with clinical benefit from first-line treatment with bevacizumab plus chemotherapy in mCRC patients.

**Electronic supplementary material:**

The online version of this article (doi:10.1186/s12876-015-0266-6) contains supplementary material, which is available to authorized users.

## Background

Access to the host vascular system and the formation of tumor blood supply represent a limitation for the progression of solid tumors. A switch to an angiogenic phenotype occurs early in tumorigenesis and is induced by hypoxia, metabolic stress, or oncogene activation. The vascular endothelial growth factor (VEGF) family of ligands and their receptors play a crucial role in tumor angiogenesis and neovascularization. VEGF stimulates proliferation of endothelial cells, vascular permeability, and attracts bone marrow-derived endothelial precursors to the tumor microenvironment. Increased levels of pro-angiogenic factors and tumor vascularization are associated with increased risk of tumor metastases and inferior survival of patients with metastatic colon cancer and other types of cancer [1].

Bevacizumab (bev) is a recombinant, humanized, monoclonal antibody that inhibits VEGF receptor signaling by binding to VEGF-A. Bevacizumab was the first angiogenic inhibitor shown to prolong survival in advanced cancer [2]. Bev in combination with chemotherapy significantly improves progression-free survival and overall survival in the first- and second-line treatment of metastatic colorectal cancer (mCRC) [3,4].

Currently, tumor KRAS gene status remains one of the most important predictive biomarker used in management of colorectal carcinoma. Beside its established role in anti-EGFR therapy, KRAS mutation may also affect the clinical outcome of anti-angiogenic therapy. Activating KRAS mutation in carcinoma cells may induce angiogenesis via several mechanisms. Oncogenic mutations of Ras intracellular signal transducers can increase VEGF production by tumor cells [1,5]. Moreover, activating Ras mutation resulted in up-regulation of pro-angiogenic interleukin-8 (CXCL-8) leading to recruitment of endothelial cells, tumor vascularisation and tumor growth *in vitro* [6] and more aggressive biological behavior *in vivo* [7]. Additionally, Ras signaling promoted angiogenesis through repression of anti-angiogenic thrombospondin-1 [8]. Thus, Ras oncogenes may contribute to tumor progression by both a direct effect on tumor cell proliferation as well as indirectly by facilitating tumor angiogenesis in a paracrine fashion*.*

The aim of the present observational study was to assess the role of KRAS status in mCRC patient treated in the the first-line with anti-angiogenic agent bevacizumab combined with oxaliplatin- or irinotecan-based chemotherapy in current clinical practice. Furthermore, we aimed to identify risk factors related to disease characteristics and treatment within the context of KRAS status.

## Methods

### Data collection

Data from 1622 mCRC patients with known KRAS status who received first-line bevacizumab plus FOLFOX, XELOX, FOLFIRI or XELIRI between January 2005 and April 2013 were mined from the non-interventional post-registration database collecting epidemiological and clinical data of patients with advanced and metastatic CRC on targeted treatment (CORECT registry). The protocol was approved by the independent ethics committee at each participating centre (Ethics Committee (EC) of the Ceske Budejovice Hospital, EC of the Chomutov Hospital, EC of the General University Hospital in Prague, EC of the Jihlava Hospital, EC of the Liberec Regional Hospital, EC of the Masaryk Hospital in Usti nad Labem, EC of the Masaryk Memorial Cancer Institute in Brno, EC of the Na Bulovce Hospital in Prague, EC of the Na Homolce Hospital in Prague, EC of the Novy Jicin Hospital, EC of the Pardubice Regional Hospital, EC of the St. Anne’s University Hospital (UH) in Brno, EC of the Thomayer Hospital in Prague, EC of the Tomas Bata Regional Hospital in Zlin, EC of the UH Brno, EC of the UH Hradec Kralove, EC of the UH in Motol, Prague, EC of the UH Olomouc, EC of the UH Ostrava, EC of the UH Pilsen) and complied with the International Ethical Guidelines for Biomedical Research Involving Human Subjects, Good Clinical Practice guidelines, the Declaration of Helsinki, and local laws. The CORECT database includes data of approximately 96% of all mCRC patients treated with targeted therapies in the Czech Republic. The security of individual records is guaranteed via de-identified data collection and the system meets all valid rules on the protection of personal data. Only authorized users can access the system. No exclusion criteria were applied in data input, mining or analysis.

### Evaluated parameters

#### KRAS testing

Tumor tissue samples were tested in local referral laboratories to identify mutations in codons 12 and 13 of exon 2 of the KRAS gene using established methods according to American Society of Clinical Oncology guidelines. In the Czech Republic in 2009, 60.2% colorectal tumors were tested as wild-type KRAS (wtKRAS) and the incidence of mutation detected was as follows: G12D (11.9%), G12V (8.8%), G13D (5.8%), G12C (3.3%), G12A (2.8%), G12S (2.0%), G12R (1.0%), G13C (0.3%), G13V (0.1%) [9].

#### Treatment

Choice of chemotherapy backbone regimen was at the physicians’ discretion according to national guidelines based on European Society for Medical Oncology guidelines. The dosage of chemotherapeutic agents were determined according to the body surface area and dose reduction was recommended only in cases of severe (grade 3) adverse events to assure optimal chemotherapy dose intensity. The chemotherapy regimens were as follows: FOLFOX4 (oxaliplatin 85 mg/m^2^ IV day1; leucovorin 200 mg/m^2^ IV days 1 and 2; 5-FU bolus 400 mg/m^2^ IV days 1 and 2; 5-FU 600 mg/m^2^ IV 22-hour continuous infusion days 1 and 2 every 2 weeks), FOLFIRI (irinotecan 180 mg/m^2^ IV day 1; leucovorin 200 mg/m^2^ IV day 1 and 2; 5-FU 600 mg/m^2^ IV 22-hour continuous infusion days 1 and 2 every 2 weeks), XELOX (oxaliplatin 130 mg/m^2^ IV day 1; capecitabine 1000 mg/m^2^ twice daily PO for 14 days every 3 weeks), or XELIRI (irinotecan 250 mg/m^2^ IV day 1; capecitabine 1000 mg/m^2^ twice daily PO for 14 days every 3 weeks). Bevacizumab was administered at a dosage of 5 mg/kg IV every 2 weeks or 7.5 mg/kg IV every 3 weeks depending on the chemotherapy regimen. Dosage of bevacizumab was not reduced.

#### Clinical response and follow-up

Data were reported to the registry every 6 months. Patients’ response to treatment and tumor measurements were determined by independent evaluation by two local radiologists according RECIST version 1.0. Patients were followed-up until death or loss to follow-up.

### Statistical analyses

Standard descriptive statistics were used to characterize the sample data set. Differences in initial categorical parameters were assessed using the Pearson chi-square test. Comparisons of the subgroups in continuous variables were based on the Mann–Whitney test. Both overall survival (OS) and progression-free survival (PFS) were estimated using the Kaplan-Meier method. OS was defined as the time from bevacizumab treatment initiation to death due to any cause. PFS was defined as the time from bev treatment initiation to progression or death due to any cause. Statistical significance of the differences in Kaplan-Meier estimates was assessed using the log-rank test. For all point estimates the 95% confidence interval (95% CI) was calculated. The univariate Cox proportional hazards model was used to evaluate the influence of all potential predictive and prognostic factors on the survival measures and subsequently the multivariable Cox proportional hazards model was used to quantify the influence of KRAS status on survival in the presence of other potential predictive and prognostic factors. A level of significance α = 0.05 was used in decision on statistical significance.

## Results

### KRAS status and disease characteristics

KRAS mutations were reported in 40.6% of mCRC patients studied; the remaining 59.4% of patients had wild-type KRAS tumors. The occurrence of KRAS somatic mutation was distributed evenly between men and women. The median age of patients with KRAS mutations was two years older than that of patients without mutation (p < 0.001). The distribution of KRAS status did not differ between primary tumors located in colon or rectum. However, we observed that patients presenting with synchronous metastases were more likely to have a tumor with KRAS mutation (p = 0.049). Resectability of metastases in mutant KRAS subgroup was comparable to resectability in wtKRAS patients. KRAS mutation was not associated with multiple metastatic sites at the time of therapy initiation. Nevertheless, we observed that KRAS mutated tumors metastasized more often to lungs (p = 0.001) (Table 1).

**Table 1:** **Patients, disease and treatment characteristics**

	WT KRAS (n = 964)	KRAS mutation (n = 658)	p-value
Male, n (%)	593 (61.5)	405 (61.6)	0.999
Age at bevacizumab treatment initiation, median, (min-max)	61 yrs (22–85)	63 yrs (22–83)	<0.001
Site of primary tumor, n (%)			
Colon	593 (61.5)	398 (60.5)	0.679
Rectum	371 (38.5)	260 (39.5)	
PS at 1st line treatment initiation*, n (%)			
PS 0	378 (52.2)	264 (51.5)	0.811
PS 1	333 (46.0)	242 (47.2)	
PS 2 or PS 3	13 (1.8)	7 (1.4)	
Adjuvant chemotherapy**, n (%)	317 (32.9)	189 (28.7)	0.081
Resectability of metastases*			
Unresectable	543 (76.5)	374 (75.7)	0.695
Potentially resectable	127 (17.9)	96 (19.4)	
Resectable	40 (5.6)	24 (4.9)	
Sites of metastases at the time of the initiation of fist-line treatment*, n (%)			
Liver	518 (67.7)	350 (64.0)	0.174
Lymph nodes	235 (30.7)	143 (26.1)	0.073
Lungs	182 (23.8)	175 (32.0)	0.001
Peritoneum	137 (17.9)	111 (20.3)	0.284
Other localization	116 (15.2)	78 (14.3)	0.693
2 and more metastasis	338 (44.2)	238 (43.5)	0.822
Bevacizumab regimen in first-line treatment, n (%)			
5 mg/kg every 2 weeks	613 (63.6)	436 (66.3)	0.290
7.5 mg/kg every 3 weeks	351 (36.4)	222 (33.7)	
CT at first-line treatment initiation, n (%)			
FOLFOX	511 (53.0)	366 (55.6)	0.259
XELOX	295 (30.6)	184 (28.0)	
FOLFIRI	107 (11.1)	83 (12.6)	
XELIRI	51 (5.3)	25 (3.8)	
Reason for first-line therapy termination, n (%)			
Disease progression	534 (64.7)	342 (63.0)	0.162
Surgery	52 (6.3)	47 (8.7)	
Adverse event of bevacizumab	48 (5.8)	24 (4.4)	
Adverse event of chemotherapy	18 (2.2)	17 (3.1)	
Other reason***	173 (17.9)	113 (20.8)	
Subsequent anti-EGFR-based treatment, n (%)****			
In second line	342 (35.5)	15 (2.3)	-
In third line	245 (25.4)	8 (1.2)	
In fourth line	21 (2.2)	3 (0.5)	

M, presence of distant metastasis; PS, performance status; *PS data were available for 75% of patients in the wtKRAS subgroup and for 78% patients in the mutant KRAS subgroup. Data on metastasis sites at first-line treatment initiation was available in 79% and 83% of patients, respectively. Data on metastases resectability were available for 74% and 75% patients, respectively. **Adjuvant regimens included FUFA biweekly infusional 5-FU/LV, FOLFOX, capecitabine, and not specified adjuvant CT. ***Other reason for first-line therapy termination included lack of data availability, CR, patient refusal, and death. ****The regimen with anti-EGFR antibodies were following: FOLFOX4+ panitumumab, FOLFIRI + panitumumab, FOLFOX4 + cetuximab, FOLFIRI + cetuximab, irinotecan (250 mg/m^2^ IV every 2 weeks) + cetuximab, panitumumab or cetuximab as single agents. Schedules and dosage of chemotherapy was identical to those applied in the first-line treatment. Panitumumab was administered 6 mg/kg IV every 2 weeks, cetuximab was administered 500 mg/m^2^ IV every 2 weeks or 400 mg/m^2^ IV first infusion, then 250 mg/m^2^ IV weekly. The registry did not provide reliable data on number of patients treated subsequently with chemotherapy alone. The chemotherapeutic regimen applied without addition of anti-EGFR antibodies were FOLFOX, FOLFIRI, XELOX or XELIRI as defined in Methods section.

### Treatment characteristics and clinical outcome of bevacizumab plus chemotherapy

**Figure 1:**
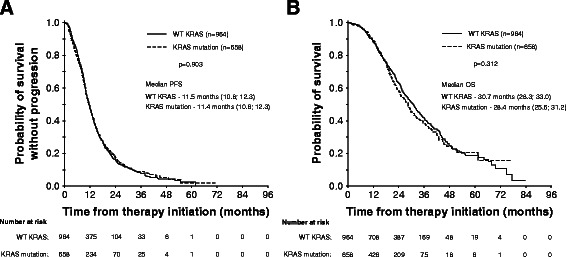
**Progression-free survival and overall survival according to KRAS mutation. A** - progression-free survival; **B** – overall survival. Progression free survival characteristics in wtKRAS vs mtKRAS subgroups were as follows: 1-year PFS 47.3 (95% CI 43.9 - 50.7) vs 47.7% (95% CI 43.5 - 51.9), 2-year PFS 15.9 (95% CI 13.2 - 18.5) vs 17.5% (95% CI 14.0 - 21.0), 3-year PFS 8.0 (95% CI 5.8 - 10.2) vs 8.9 (95% CI 6.1 - 11.8). Overall survival characteristics in wtKRAS vs mtKRAS subgroups were as follows: 1-year OS 88.1 (95% CI 85.9 - 90.3) vs 89.2% (95% CI 86.6 - 91.8), 2-year PFS 63.0 (95% CI 59.5 - 66.5) vs 58.5% (95% CI 53.8 - 63.2), 3-year PFS 41.8 (95% CI 37.8 - 45.8) vs 38.1 (95% CI 32.8 - 43.5).

There was no evident bias caused by the uneven distribution of cases with KRAS mutation in two bevacizumab schedules, chemotherapeutic regimens, or first-line treatment duration. Patients with mutated KRAS tumors achieved the best treatment response and a response rate equal to the clinical outcome of the wtKRAS subgroup (Table 1). Median PFS from treatment initiation was 11.5 months (95% CI 11.0 - 12.1). Median overall survival was 29.5 months (95% CI 27.8 - 31.2). The bevacizumab regimen every 2 versus every 3 weeks resulted in comparable PFS and OS (data not shown). Median PFS was 11.5 months in wtKRAS patients vs 11.4 months in mtKRAS subgroup (Figure 1A) and median OS was in wtKRAS patients 30.7 months over 28.4 months in patients with KRAS mutation (Figure 1B).

### Toxicity related to first-line treatment with bevacizumab

The most frequent adverse events considered to be related to bevacizumab were thromboembolic complication in 58 (3.6%) patients, hypertension in 53 (3.3%), proteinuria in 26 (1.6%), bleeding in 20 (1.2%), and GIT perforation in 6 (0.3%) patients. The grade 3 or 4 adverse events related to bevacizumab had the following occurrence: thromboembolic disease in 20 (1.2%) patients, hypertension in 18 (1.1%), bleeding in 5 (0.3%), GIT perforation in 2 (0.1%), and proteinuria in 1 (0.1%). KRAS status had no influence on the incidence of adverse events, their severity (data not shown) or termination of therapy for toxicity (Table 1).

### Factors affecting clinical outcome of treatment for mCRC

**Figure 2:**
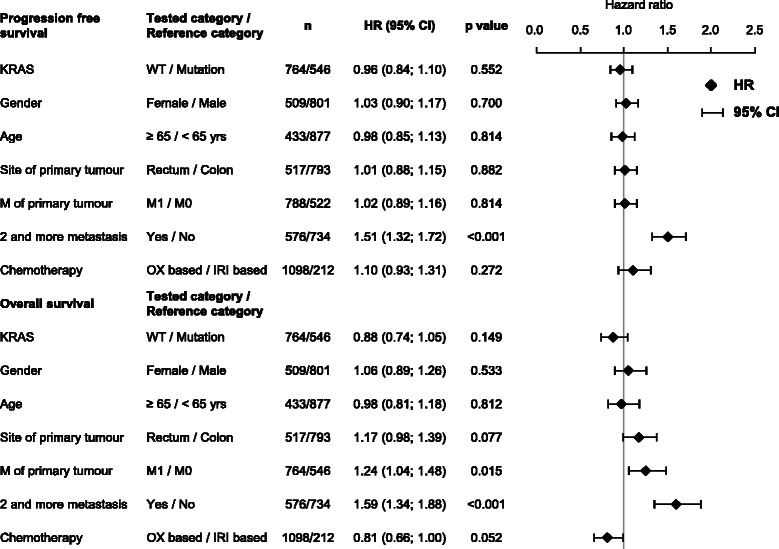
**Results of multivariable Cox analysis for progression-free survival and overall survival.** OX-based, FOLFOX or XELOX; IRI-based, FOLFIRI or XELIRI.

Multivariable Cox analyses (Figure 2) confirmed risk factors affecting the outcome from first-line treatment with bev plus chemotherapy observed in univariate Cox analysis (data not shown). Beside KRAS status, progression free survival was independent of gender, age, site of primary tumor, presence of synchronous metastasis, and chemotherapy regimen (Figure 2). Presence of multiple metastatic sites at the time of therapy initiation was the risk factor for early progression (HR = 1.51; p < 0.001) and this risk was higher in patients with KRAS mutation (HR = 1.77; 95% CI 1.43 - 2.18) compared to patients with wtKRAS (HR = 1.37; 95% CI 1.16 - 1.62). Overall survival tended to be improved in patients treated in the first line with bevacizumab with oxaliplatin compared to irinotecan-based chemotherapy (HR = 0.81, p = 0.052). Patients presenting with synchronous metastases had shorter overall survival (HR = 1.24; p = 0.015) and in the subgroup analysis, the significance of this effect was limited to wtKRAS subgroup (HR = 1.25; p = 0.020). Similarly to PFS, presence of multiple metastatic sites was the risk factor for shorter overall survival (HR = 1.59; p < 0.001).

**Figure 3:**
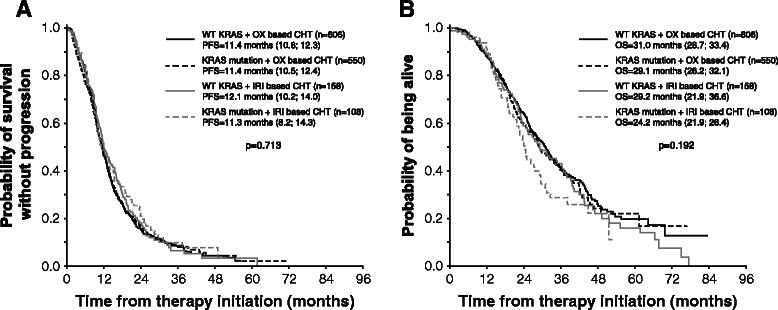
**Progression-free survival and overall survival according to KRAS mutation and type of chemotherapy. A** - progression-free survival; **B** – overall survival.

KRAS subgroup analysis of clinical outcome in the context of chemotherapy backbone confirmed similar PFS in patients treated with bev/OX-based or bev/IRI-based with or without KRAS mutation (p = 0.716; Figure 3A). In patients with wtKRAS tumors, median OS was 31.0 months in bev/OX-based subgroup and 29.2 months in case of bev/IRI-based first-line treatment. In patients with KRAS mutation, median OS was 29.1 months in bev/OX-based treatment and 24.2 months in bev/IRI-based subgroup (p = 0.192, Figure 3B). Thus, the trend toward relatively improved overall survival in patients who started with bev plus XELOX or FOLFOX may be explained by shorter OS in KRAS mutant patients who received anti-angiogenic therapy on an irinotecan backbone.

**Figure 4:**
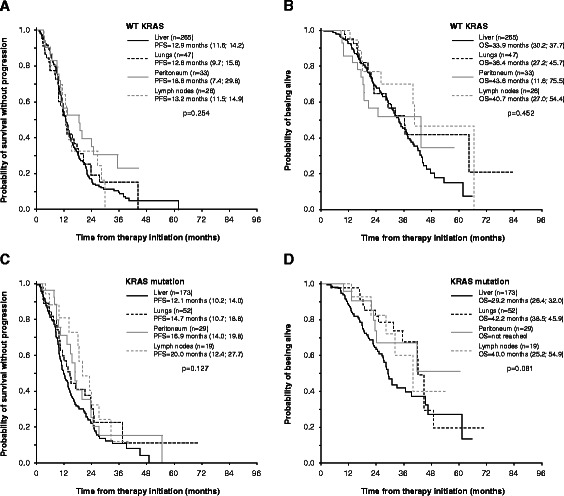
**Progression-free survival and overall survival according to KRAS mutation and metastatic site in patients with metastatic disease limited to one distant organ. A**, **C** – progression-free survival; **B**, **D** – overall survival.

KRAS subgroup analysis of clinical outcome in the context of location of metastatic disease revealed no difference in PFS between patients with only hepatic wtKRAS metastatic involvement (median PFS 12.9 months; 95% CI 11.6 - 14.2) compared to mtKRAS/hepatic subgroup (median PFS 12.1 months; 95% CI 10.2 - 14.0; p = 0.956). Similarly, median OS was 33.9 months (95% CI 30.2 - 37.7) in wtKRAS/hepatic subgroup and 29.2 months (95% CI 26.4 - 32.0) in mtKRAS/hepatic subgroup (p = 0.542). Similarly, the difference in PFS or OS between wtRAS and mtKRAS was not observed for metastatic involvement of lungs, peritoneum or lymph nodes (Figure 4). Location of metastasis in single organ involvement was also not associated with effect on PFS or OS (Figure 4).

## Discussion

The present data demonstrates that mCRC patients treated with bevacizumab plus combination chemotherapy have similar outcomes regardless of KRAS mutation status. Currently, standard first-line treatment for patients with mCRC includes combination chemotherapy with either an anti-EGFR agent (cetuximab or panitumumab), or bevacizumab. Adding bevacizumab to chemotherapy prolongs survival in advanced colorectal cancer patients. However, not all mCRC patients respond to this treatment and identification of a subgroup of patients who will benefit most from bevacizumab currently represents a challenge. Data on the prognostic and/or predictive significance of KRAS status in mCRC patients treated with first-line bevacizumab-containing regimens were obtained mostly as secondary endpoints in trials comparing bevacizumab vs anti-EGFR therapy efficacy (see Additional file 1: Table S1). In general, these studies failed to demonstrate KRAS status as a predictive factor for anti-VEGF therapy in the first-line treatment of mCRC, consistent with the present study. The results of the MACRO trial indicated that KRAS status may be a prognostic factor in mCRC patients treated with bevacizumab; however, that study setting did not allow the evaluation of the predictive role of KRAS status [10]. Evaluation of OS in the studies evaluating the prognostic role of KRAS in patients treated with first-line bevacizumab, including the present study (summarized in Additional file 1: Table S1), is difficult because the patients’ survival is influenced by subsequent line of therapy and patients with wtKRAS tumors could benefit often from anti-EGFR therapy after progression on bev/chemotherapy. Accordingly, the insignificant prolongation of overall survival by 2.3 months in wtKRAS patients over the mutated KRAS subgroup may be to some extent attributed to subsequent anti-EGFR therapy (Table 1).

The adverse events from bevacizumab treatment observed in this study are consistent with those published in post-registrational trials, including BEAT, BRiTE and ARIES [11-13]. We confirmed that severe (grade 3–5) events, including arterial thromboembolic events, proteinuria, gastrointestinal perforation and wound-healing complications were rare with incidence and spectrum of adverse events comparable across all age groups [14]. Taken together, the adverse events related to bevacizumab are acceptable and well tolerated.

Prospective data on the optimal sequence of chemotherapy regimen used with targeted therapy of metastatic colorectal cancer is lacking. It has been demonstrated that first-line regimens combining fluoropyrimidines with oxaliplatin or irinotecan are of comparable efficacy [15,16]. Accordingly, the optimal choice for the chemotherapy to be used in combination with bevacizumab in the initial treatment of mCRC should be based on the characteristics of the patient and tumor, as well as the drug efficacy and toxicity profile. FOLFOX or XELOX is considered as a convenient first-line treatment option for mCRC patients as they cause less diarrhea, nausea, and alopecia than FOLFIRI or XELIRI, despite a potential for dose-limiting neurotoxicity. In the present study, patients who received in the first line bev/OX-based chemotherapy tended to have longer overall survival over those treated with irinotecan-based chemotherapy with bevacizumab. As we did not observe a difference in performance status (ECOG of 0 or 1) between patients who started treatment with bev/OX versus bev/IRI-based therapy in the first-line setting (data not shown), the trend toward better OS in bev/OX subgroup is unlikely to be attributed to better performance status in these patients. Interestingly, the inferior efficacy of treatment with the irinotecan backbone was restricted to the subgroup of patients with KRAS mutation, with median OS in the bev/OX-based subgroup being 5 months longer than in the bev/IRI-based treatment. Based on *in vitro* experiments showing that KRAS mutation is a predictor of oxaliplatin sensitivity in colon cancer cells, it has been suggested that mutant KRAS CRC patients might benefit more from receiving first-line oxaliplatin-based regimens [17]. Although we have not observed a better response in KRAS mutated mCRC patients treated with bev/OX-based therapy over those with wtKRAS, as it was reported when patients with advanced CRC were treated in the first line with FOLFOX-6 [18], the reported sensitivity of mutated KRAS carcinoma cells to oxaliplatin may explain the improved clinical outcome when KRAS mutant patients were treated with bev/OX-based over bev/IRI-based therapy.

We have confirmed that patients presenting with synchronous metastases have an inferior prognosis compared to patients with metachronous metastases. Among the evaluated parameters, metastatic involvement of multiple organs at time of treatment initiation was the strongest prognostic factor reducing both PFS and OS. Within the subgroup of patients with metastatic disease limited to one distant organ we did not observed difference in clinical outcome with regard to hepatic or extrahepatic (including pulmonary) involvement. Interestingly, together with Rossi *et al.* [19], we observe trend toward longer both PFS and OS in patients with extrahepatic disease and KRAS mutation than in wtKRAS subgroup when treated with bevacizumab plus chemotherapy. Apart from this fact, we and others [20-22] have reported that KRAS mutation in colorectal cancer itself is associated with pulmonary metastasis. Findings from the VICTOR trial showing that KRAS mutant tumors are associated with an increased risk of lung relapse in CRC patients supported the role of chest imaging in surveillance of colorectal cancer patients, particularly of those with resected primary mutated KRAS carcinoma [20,22]. The reason for increased incidence of lung metastases in KRAS mutated colorectal tumors remains unknown at this moment. Considering the decreased proportion of lymph node metastasis in mutated KRAS patients compared to wtKRAS subgroup (Table 1, [23,24]), it seems that carcinoma cells with activating mutation in KRAS may exhibit a more hematogenous metastatic spread rather than along a lymphogenous path. Survival of tumor cells within the bloodstream and adhesion in the vasculature at the metastatic sites depend on tumor cell – platelet interactions [25]. We hypothesize that activating mutation of KRAS inducing expression of molecules responsible for interaction with platelets, such as tissue factor [26], cyclooxygenase and metalloproteinase-9 [27], or cathepsin B [28] might contribute to increased protection of these carcinoma cells against shear stress as well as to enhanced adhesion properties which in turn leads to onset of pulmonary metastasis of mutated KRAS carcinoma cells and higher metastatic activity in general [29].

The present study is a retrospective analysis, and thus an unintentional selection bias for a subset of patients is possible. However, the parameters of our analysis that confer substantial reliability to the presented results are, for example, unselective multicenter input of evaluated data and the proportion of tumors with wtKRAS vs mtKRAS, mirroring the proportion of KRAS mutation previously detected in the Czech Republic during a one year survey [9]. We excluded a potential bias caused by inclusion patients with known KRAS status only, as no difference was observed between PFS of mCRC patients treated in the first-line with bevacizumab and OX- or IRI-based chemotherapy with KRAS-known (11.5 months, 95% CI 11.0-12.1) and KRAS-unknown (11.6 months, 95% CI 11.0-12.2) which is further underlined by KRAS-unselected published data on first-line bevacizumab in mCRC patients based on CORECT registry [14]. An existing limitation of the present study is that we had no data specifying the type of KRAS mutation, NRAS mutation or data on BRAF mutation. Mutation of BRAF and KRAS are, in the vast majority cases, mutually exclusive, and thus, the wtKRAS subgroup in the present study included an unknown number of BRAF mutated cases who might have worse survival rates. However, estimating from our findings of BRAF mutations in 3.8% of mCRC patients (unpublished data from diagnostic testing), contamination of the wtKRAS subgroup with mutant BRAF patients is unlikely to have been substantial. Regarding specific mutations in the KRAS gene, there is emerging data showing that mutation in codon 13 leads to a more aggressive disease course with extensive synchronous metastases compared to colorectal carcinoma with mutation in codon 12 [30]. Mutation in codon 13 is reported to confer worse prognosis and outcome than alteration in codon 12 [31]. On the other hand, when treated with cetuximab and chemotherapy, patients with mutation in codon 13 demonstrated a trend toward longer PFS than the codon 12-mutated subgroup but also a better outcome than patients with tumors without KRAS mutation [30,31]. KRAS codon 12 (Cys12 = G12C) or codon 13 (Asp13 = G13D) mutation resulted in different vascular strategy *in vitro*; the neovascularization in Asp13 cells was associated with high levels of VEGF-A but less effective vasculature compared to their Cys12 counterpart [32]. Moreover, a trend toward increased vascular invasion in codon 13-mutated tumors has been observed in colorectal cancer patients [33]. Thus, the different biological role of KRAS mutation in codon 13 may have also implications for anti-angiogenic treatment with bevacizumab.

## Conclusion

The present study demonstrates that KRAS mutation in codon 12 or 13 does not interfere with clinical benefit from bevacizumab plus chemotherapy in patients with mCRC during first- line therapy. We speculate that this finding reflects specific aspects of mutated KRAS tumor biology rather than an irrelevance of KRAS in bev/chemotherapy outcome. One factor might be the relative sensitivity of KRAS-mutated carcinoma cells to oxaliplatin. Another factor might be enhanced pro-angiogenic properties of CRC cells with KRAS mutation which, however, might be restricted to certain KRAS mutation subtypes [32,34]. These cancers might be somewhat more “angiogenesis-dependent” and therefore more sensitive to VEGF withdrawal caused by bevacizumab. Similarly, a high expression of VEGF-A and VEGFR-2 predicted improved PFS in metastatic breast cancer patients treated with docetaxel/bevacizumab [35]. Taken together, it is becoming clear that establishment of a predictive role of KRAS mutation for treatment of mCRC with chemotherapy and biological agents, including bevacizumab, cannot be elucidated without evaluation of specific KRAS mutations.

## Additional file

Additional file 1: Table S1. Results of first-line bevacizumab and chemotherapy treatment in phase III trials or large-scale studies of metastatic colorectal cancer patients with wt versus mutated KRAS.

## Abbreviations

bev: Bevacizumab
CI: Confidence interval
CRC: Colorectal carcinoma
EC: Ethics committee
ECOG: Eastern Cooperative Oncology Group
EGFR: Epidermal growth factor receptor
GIT: Gastrointestinal tract
HR: Hazard ratio
IRI: Irinotecan
KRAS: Kirsten rat sarcoma viral oncogene homolog
mCRC: Metastatic colorectal cancer
mtKRAS: Mutant KRAS
OS: Overall survival
OX: Oxaliplatin
PFS: Progression-free survival
RECIST: Response evaluation criteria in solid tumors
UH: University Hospital
VEGF: Vascular endothelial growth factor
VEGFR: Vascular endothelial growth factor receptor
wtKRAS: Wild-type KRAS
