# Supplementary material for: Efficacy of bevacizumab and chemotherapy in the first-line treatment of metastatic colorectal cancer: broadening KRAS-focused clinical view
Source: BMC Gastroenterol. 2015 Mar 24;15:37. doi: 10.1186/s12876-015-0266-6 (PMC4376345; doi:10.1186/s12876-015-0266-6)
Supplement: Additional file 1: Table S1. — Results of first-line bevacizumab and chemotherapy treatment in phase III trials or large-scale studies of metastatic colorectal cancer patients with wt versus mutated KRAS. [file 12876_2015_266_MOESM1_ESM.doc]

| **Trial (reference)** | **Treatment** | **Number of patients KRAS WT/MT (%)** | **PFS KRAS WT/MT (months)** | **OS KRAS WT/MT (months)** |
| --- | --- | --- | --- | --- |
| AVF2107g | B+IFL | 85/44 (66/34) | 13.5 vs 9.3; p=NS | 27.7 vs 19.9; p=NA |
| PACCE | B+oxaliplatin-based CT | 261/164 (61/39) | 11.5 vs 11.0; p=NA | 24.5 vs 19.3 p=NA |
|  | B+irinotecan-based CT |  | 12.5 vs 11.9 p=NA | 19.8 vs 20.5 p=NA |
| AGITG MAX | B+Capecitabine+/-mitomycin | 224/90 (71/29) | 8.8 vs 8.2; p=NS | 19.8 vs 17.6; p=NS |
|  | B+mFOLFOX6 | 49/36 (58/42) | 10.9 vs 12.1; p=NA | 18.8 vs 22.4; p=NA |
| CAIRO-2 | B+CAPOX | 156/108 (59/41) | 10.6 vs 12.5; p=0.80 | 22.4 vs 24.9; p=0.82 |
| MACRO | B+XELOX | 219/175 (56/44) | 10.9 vs 9.2; p=0.0038 | 26.7 vs 18.4; p=0.0002 |
| FIRE-3 | B+FOLFIRI | 334/62 | 10.4 vs 12.2; p=NA | 25.9 vs 20.6; p=NA |
| Present analysis | B+oxaliplatin-based CT | 964/658 | 11.4 vs 11.4; p=0.970 | 31.0 vs 29.1; p=0.558 |
|  | B+irinotecan-based CT |  | 12.1 vs 11.3; p=0.704 | 29.2 vs 24.2; p=0.212 |

Table S1. Results of first-line bevacizumab and chemotherapy treatment **in phase III trials** or large-scale studies of metastatic colorectal cancer patients with wt versus mutated KRAS.

Abbreviations: 5-FU/LV, 5-fluorouracil and leucovorin; mFOLFOX6,modified infusional 5-FU/LV with oxaliplatin; IFL, bolus 5-FU/LV with irinotecan; mCRC, metastatic colorectal cancer; OS, overall survival; PFS, progression-free survival; CAPOX, XELOX, capecitabine with oxaliplatin; B, bevacizumab; CT, chemotherapy; NA, not available; NS, not significant

References:

1. Hurwitz HI, Yi J, Ince W, Novotny WF, Rosen O: **The clinical benefit of bevacizumab in metastatic colorectal cancer is independent of K-ras mutation status: analysis of a phase III study of bevacizumab with chemotherapy in previously untreated metastatic colorectal cancer.** *Oncologist* 2009, **14:**22-28.

2. Hecht JR, Mitchell E, Chidiac T, Scroggin C, Hagenstad C, Spigel D, Marshall J, Cohn A, McCollum D, Stella P, et al: **A randomized phase IIIB trial of chemotherapy, bevacizumab, and panitumumab compared with chemotherapy and bevacizumab alone for metastatic colorectal cancer.** *J Clin Oncol* 2009, **27:**672-680.

3. Price TJ, Hardingham JE, Lee CK, Weickhardt A, Townsend AR, Wrin JW, Chua A, Shivasami A, Cummins MM, Murone C, Tebbutt NC: **Impact of KRAS and BRAF Gene Mutation Status on Outcomes From the Phase III AGITG MAX Trial of Capecitabine Alone or in Combination With Bevacizumab and Mitomycin in Advanced Colorectal Cancer.** *J Clin Oncol* 2011, **29:**2675-2682.

4. Saltz L, Badarinath S, Dakhil S, Bienvenu B, Harker WG, Birchfield G, Tokaz LK, Barrera D, Conkling PR, O'Rourke MA, et al: **Phase III trial of cetuximab, bevacizumab, and 5-fluorouracil/leucovorin vs. FOLFOX-bevacizumab in colorectal cancer.** *Clin Colorectal Cancer* 2012, **11:**101-111.

5. Tol J, Koopman M, Cats A, Rodenburg CJ, Creemers GJ, Schrama JG, Erdkamp FL, Vos AH, van Groeningen CJ, Sinnige HA, et al: **Chemotherapy, bevacizumab, and cetuximab in metastatic colorectal cancer.** *N Engl J Med* 2009, **360:**563-572.

6. Diaz-Rubio E, Gomez-Espana A, Massuti B, Sastre J, Reboredo M, Manzano JL, Rivera F, Safont MJ, Montagut C, Gonzalez E, et al: **Role of Kras status in patients with metastatic colorectal cancer receiving first-line chemotherapy plus bevacizumab: a TTD group cooperative study.** *PLoS One* 2012, **7:**e47345.

7. Stintzing S, Jung A, Rossius L, Modest DP, Fischer von Weikersthal L, Decker T, Möhler M, Scheithauer W, Kirchner T, Heinemann V: **Analysis of KRAS/NRAS and BRAF mutations in FIRE-3: A randomized phase III study of FOLFIRI plus cetuximab or bevacizumab as first-line treatment for wild-type (WT)KRAS (exon2) metastatic colorectal cancer (mCRC) patients.** *Eur J Cancer* 2013, **49:**LBA17.
